# Supplementary material for: Organic farming expansion drives natural enemy abundance but not diversity in vineyard‐dominated landscapes
Source: Ecol Evol. 2019 Nov 14;9(23):13532–42. doi: 10.1002/ece3.5810 (PMC6912908; doi:10.1002/ece3.5810)

**Supporting information: Organic farming expansion drives natural enemy abundance not diversity in agricultural landscapes**

Lucile Muneret, Arthur Auriol, Olivier Bonnard, Sylvie Richart-Cervera, Denis Thiéry & Adrien Rusch

**TABLE S1** Responses of the natural enemy abundance, richness and evenness to environmental conditions in vineyards for the community sampled in the foliage. For each response variable, we recalculated the Akaike weights among all of the models from the four different sets ('M0', 'M1' and both 'M2' obtained using multimodel inference). We, therefore, estimated the relative importance of each level of complexity for a given response variable that gave us the "best scale" of response (Figure S3). The sum of the Akaike weights ("Sum Wi") of the models obtained at the best scale provided the model’s probability of being the top model across all of the scales. Other parameters reported in this table come from models M0, M1 and M2 at the best scale for each response variable. For a given response variable, if the M0 model had a higher “Sum Wi” than M1 and M2, we only wrote M0 outputs in this table. R² marginal and R² conditional are reported. R2 values were calculated using the best models at the best scale. The standard deviation of the random terms are reported. Estimates, confident interval (2,5 - 97.5%) and relative importance variable were reported for each predictor. “S.D. Random term” has been obtained based on the best model at a given scale. Values in bold are significant (confident interval did not include zero and relative variable importance equal to 1).

| **Response variable** | **Model with R² marginal and R² conditional** | **Sum Wi** | **AIC** | **Explanatory variables selected and random term** | **Estimates** | **Confidence intervals** | **Relative variable importance** | **S.D Random term** |
| --- | --- | --- | --- | --- | --- | --- | --- | --- |
| **Total abundance** | **M0*** |  |  |  |  |  |  |  |
|  | (R²m = 0.05; | 0 | 513,91 | Intercept | 476,74 | (387.79; 565.20) | - |  |
|  | R²c = 0.19) |  |  | Field age | -67,14 | (-264.43; 130.60) | 1 |  |
|  |  |  |  | Vine stock density | 24,09 | (-193.86; 242.64) | 1 |  |
|  |  |  |  | Total TFI | -20,08 | (-189.85; 159.27) | 1 |  |
|  |  |  |  | Tillage intensity | -30,91 | (-216.57; 152.09) | 1 |  |
|  |  |  |  | Crop productivity | 95,80 | (-113.81; 305.66) | 1 |  |
|  |  |  |  | *pair* |  |  |  | *107,8* |
|  |  |  |  |  |  |  |  |  |
|  | **M1*** |  |  |  |  |  |  |  |
|  | (R²m = 0.03; | 0,00 | 547,44 | Intercept | 433,58 | (316.61; 550.40) | - |  |
|  | R²c = 0.11) |  |  | Local farming system (:Conventional) | 86,04 | (-76.02; 247.40) | 1 |  |
|  |  |  |  | *pair* |  |  |  | *78,54* |
|  |  |  |  |  |  |  |  |  |
|  | **M2 at the 500-m scale*** |  |  |  |  |  |  |  |
|  | (R²m = 0.12; | 0,74 | 499,68 | Intercept | 428,70 | (315.55; 542.52) | - |  |
|  | R²c = 0.21) |  |  | Local farming system (:Conventional) | 73,70 | (-81.92; 226.65) | 1 |  |
|  |  |  |  | % organic farming | 41,04 | (-184.41; 266.89) | 1 |  |
|  |  |  |  | % semi-natural habitats | -77,96 | (-341.36; 186.99) | 1 |  |
|  |  |  |  | Local farming system : % organic farming | 195,24 | (-127.80; 518.94) | 1 |  |
|  |  |  |  | Local farming system : % semi-natural habitats | 177,87 | (-157.23; 494.26) | 1 |  |
|  |  |  |  | % organic farming : % semi-natural habitats | -83,74 | (-506.53; 336.10) | 1 |  |
|  |  |  |  | *pair* |  |  |  | *87,99* |
|  |  |  |  |  |  |  |  |  |
| **Spider abundance** | **M0*** |  |  |  |  |  |  |  |
|  | (R²m = 0.20; | 0,00 | 402,14 | Intercept | 171,66 | (147.17; 195.83) | - |  |
|  | R²c = 0.75) |  |  | Field age | -4,96 | (-40.63; 30.59) | 1 |  |
|  |  |  |  | Vine stock density | -11,78 | (-56.24; 31.80) | 1 |  |
|  |  |  |  | **Total TFI** | **-60,09** | **(-86.35; -33.83)** | **1** |  |
|  |  |  |  | **Tillage intensity** | **-37,49** | **(-68.59; -6.38)** | **1** |  |
|  |  |  |  | Crop productivity | -0,42 | (-39.49; 38.33) | 1 |  |
|  |  |  |  | *pair* |  |  |  | *51,17* |
|  |  |  |  |  |  |  |  |  |
|  | **M1*** |  |  |  |  |  |  |  |
|  | (R²m = 0.22; | 0,00 | 409,49 | Intercept | 155,73 | (124.39; 187.47) | - |  |
|  | R²c = 0.74) |  |  | **Total TFI** | **-40,64** | **(-77.26; -3.71)** | **1** |  |
|  |  |  |  | **Tillage intensity** | **-53,86** | **(-91.09; -16.80)** | **1** |  |
|  |  |  |  | Local farming system (:Conventional) | 32,59 | (-11.41; 76.99) | 1 |  |
|  |  |  |  | *pair* |  |  |  | *47,87* |
|  |  |  |  |  |  |  |  |  |
|  | **M2 at the 1000-m scale*** |  |  |  |  |  |  |  |
|  | (R²m = 0.41; | 0,78 | 375,36 | Intercept | 153,02 | (124.59; 181.79) | - |  |
|  | R²c = 0.75) |  |  | **Total TFI** | **-41,28** | **(-77.20; -5.03)** | **1** |  |
|  |  |  |  | **Tillage intensity** | **-56,08** | **(-91.18; -21.42)** | **1** |  |
|  |  |  |  | Local farming system (:Conventional) | 34,13 | (-7.70; 76.75) | 1 |  |
|  |  |  |  | % organic farming | -19,19 | (-63.69; 26.13) | 1 |  |
|  |  |  |  | % semi-natural habitats | 27,72 | (-38.46; 95.63) | 1 |  |
|  |  |  |  | Local farming system : % organic farming | -20,85 | (-64.70; 23.25) | 1 |  |
|  |  |  |  | Local farming system : % semi-natural habitats | 1,41 | (-41.44; 44.32) | 1 |  |
|  |  |  |  | % organic farming : % semi-natural habitats | -37,09 | (-144.18; 72.10) | 1 |  |
|  |  |  |  | *pair* |  |  |  | *40,6* |
|  |  |  |  |  |  |  |  |  |
| **Ant abundance** | **M0** |  |  |  |  |  |  |  |
|  | (R²m = 0; | 0,25 | 130,67 | Intercept | 5,00 | (4.60; 5.39) | - |  |
|  | R²c = 0.15) |  |  | Crop productivity | 0,11 | (-0.37; 1.09) | 0,3 |  |
|  |  |  |  | *pair* |  |  |  | *0,44* |
|  |  |  |  |  |  |  |  |  |
|  | **M1*** |  |  |  |  |  |  |  |
|  | (R²m = 0; | 0,08 | 130,67 | Intercept | 5,00 | (4.61; 5.38) | - |  |
|  | R²c = 0.15) |  |  | Local farming system (:Conventional) |  |  | 0 |  |
|  |  |  |  | *pair* |  |  |  | *0,44* |
|  |  |  |  |  |  |  |  |  |
|  | **M2 at the 500-m scale** |  |  |  |  |  |  |  |
|  | (R²m = 0.08; | 0,38 | 130,45 | Intercept | 5,00 | (4.62; 5.38) | - |  |
|  | R²c = 0.20) |  |  | Local farming system (:Conventional) |  |  | 0 |  |
|  |  |  |  | % organic farming | 0,24 | (-0.21; 1.32) | 0,43 |  |
|  |  |  |  | % semi-natural habitats | -0,28 | (-1.37; 0.18) | 0,47 |  |
|  |  |  |  | Local farming system : % organic farming |  |  | 0 |  |
|  |  |  |  | Local farming system : % semi-natural habitats |  |  | 0 |  |
|  |  |  |  | % organic farming : % semi-natural habitats |  |  | 0 |  |
|  |  |  |  | *pair* |  |  |  | *0,4* |
|  |  |  |  |  |  |  |  |  |
| **Earwig abundance** | **M0** |  |  |  |  |  |  |  |
|  | (R²m = 0.04; | 0,43 | 145,18 | Intercept | 1,43 | (0.86; 2.00) | - |  |
|  | R²c = 0.57) |  |  | Field age |  |  | 0 |  |
|  |  |  |  | Vine stock density | 0,14 | (-0.44; 1.43) | 0,27 |  |
|  |  |  |  | Total TFI | 0,24 | (-0.10; 1.31) | 0,39 |  |
|  |  |  |  | Tillage intensity | -0,06 | (-1.22; 0.31) | 0,13 |  |
|  |  |  |  | Crop productivity | 0,04 | (-0.48; 1.20) | 0,1 |  |
|  |  |  |  | *pair* |  |  |  | *1,04* |
|  |  |  |  |  |  |  |  |  |
| **Lacewing abundance** | **M0** |  |  |  |  |  |  |  |
|  | (R²m = 0.08; | 0,06 | 245,32 | Intercept | 6,27 | (4.53; 8.00) | - |  |
|  | R²c = 0.13) |  |  | Field age | -0,41 | (-4.68; 3.30) | 0,6 |  |
|  |  |  |  | Vine stock density | -2,29 | (-6.90; 1.54) | 0,85 |  |
|  |  |  |  | Total TFI | 0,20 | (-3.16; 3.93) | 0,53 |  |
|  |  |  |  | Tillage intensity | -0,03 | (-3.75; 3.62) | 0,53 |  |
|  |  |  |  | Crop productivity | 2,95 | (-1.21; 7.11) | 1 |  |
|  |  |  |  | *pair* |  |  |  | *1,21* |
|  |  |  |  |  |  |  |  |  |
|  | **M1** |  |  |  |  |  |  |  |
|  | (R²m = <0,01; | 0,00 | 249,63 | Intercept | 6,32 | (4.18; 8.46) | - |  |
|  | R²c = 0.09) |  |  | Local farming system (:Conventional) | -0,11 | (-3.46; 3.04) | 0,54 |  |
|  |  |  |  | *pair* |  |  |  | *1,62* |
|  |  |  |  |  |  |  |  |  |
|  | **M2 at the 500-m scale*** |  |  |  |  |  |  |  |
|  | (R²m = 0.16; | 0,48 | 237,66 | Intercept | 6,33 | (4.18; 8.47) | - |  |
|  | R²c = 0.29) |  |  | Local farming system (:Conventional) | -0,44 | (-3.29; 2.39) | 1 |  |
|  |  |  |  | % organic farming | 3,74 | (-0.53; 8.01) | 1 |  |
|  |  |  |  | % semi-natural habitats | -0,96 | (-5.93; 4.01) | 1 |  |
|  |  |  |  | Local farming system : % organic farming | -4,94 | (-10.90; 1.04) | 1 |  |
|  |  |  |  | Local farming system : % semi-natural habitats | 4,18 | (-1.84; 10.13) | 1 |  |
|  |  |  |  | % organic farming : % semi-natural habitats | -3,55 | (-11.62; 4.70) | 1 |  |
|  |  |  |  | *pair* |  |  |  | *2,01* |
|  |  |  |  |  |  |  |  |  |
| **Harvestman abundance** | **M0*** |  |  |  |  |  |  |  |
|  | (R²m = 0.15; | 0,00 | 351,37 | Intercept | 48,07 | (37.42; 58.89) | - |  |
|  | R²c = 0.66) |  |  | Field age | 1,33 | (-16.49; 19.21) | 1 |  |
|  |  |  |  | **Vine stock density** | **25,90** | **(4.41; 46.76)** | **1** |  |
|  |  |  |  | Total TFI | 1,50 | (-11.82; 14.72) | 1 |  |
|  |  |  |  | Tillage intensity | 2,54 | (-13.07; 18.09) | 1 |  |
|  |  |  |  | Crop productivity | -9,67 | (-28.66; 9.57) | 1 |  |
|  |  |  |  | *pair* |  |  |  | *21,74* |
|  |  |  |  |  |  |  |  |  |
|  | **M1*** |  |  |  |  |  |  |  |
|  | (R²m = 0,12; | 0,00 | 361,34 | Intercept | 45,09 | (33.23; 57.08) | - |  |
|  | R²c = 0.67) |  |  | **Vine stock density** | **18,09** | **(1.84; 34.36)** | **1** |  |
|  |  |  |  | Local farming system (:Conventional) | 6,11 | (-4.53; 17.03) | 1 |  |
|  |  |  |  | *pair* |  |  |  | *21,76* |
|  |  |  |  |  |  |  |  |  |
|  | **M2 at the 1000-m scale*** |  |  |  |  |  |  |  |
|  | (R²m = 0.29; | 0,87 | 333,91 | Intercept | 45,86 | (34.77; 57.08) | - |  |
|  | R²c = 0.72) |  |  | **Vine stock density** | **15,71** | **(0.78; 30.75)** | **1** |  |
|  |  |  |  | Local farming system (:Conventional) | 7,06 | (-2.62; 17.64) | 1 |  |
|  |  |  |  | **% organic farming** | **-23,01** | **(-44.79; -0.97)** | **1** |  |
|  |  |  |  | % semi-natural habitats | -16,83 | (-49.92; 16.03) | 1 |  |
|  |  |  |  | Local farming system : % organic farming | 11,80 | (-9.53; 33.79) | 1 |  |
|  |  |  |  | Local farming system : % semi-natural habitats | 19,79 | (-1.19; 40.69) | 1 |  |
|  |  |  |  | % organic farming : % semi-natural habitats | 24,41 | (-28.09; 76.24) | 1 |  |
|  |  |  |  | *pair* |  |  |  | *20,16* |
|  |  |  |  |  |  |  |  |  |
| **Total species richness abundance** | **M0** |  |  |  |  |  |  |  |
|  | (R²m = 0.04; | 0,05 | 260,97 | Intercept | 17,49 | (15.33; 19.65) | - |  |
|  | R²c = 0.07) |  |  | Field age | 1,39 | (-3.03; 6.55) | 0,79 |  |
|  |  |  |  | Vine stock density | -0,12 | (-5.41; 5.05) | 0,7 |  |
|  |  |  |  | Total TFI | -1,27 | (-6.15; 2.84) | 0,76 |  |
|  |  |  |  | Tillage intensity | 0,72 | (-3.68; 5.76) | 0,7 |  |
|  |  |  |  | Crop productivity | -0,52 | (-5.77; 4.27) | 0,7 |  |
|  |  |  |  | *pair* |  |  |  | *1,32* |
|  |  |  |  |  |  |  |  |  |
|  | **M1** |  |  |  |  |  |  |  |
|  | (R²m = <0,01; | 0,00 | 265,07 | Intercept | 17,30 | (14.64; 19.95) | - |  |
|  | R²c = <0,01) |  |  | Local farming system (:Conventional) | 0,39 | (-3.51; 4.79) | 0,61 |  |
|  |  |  |  | *pair* |  |  |  | *0,42* |
|  |  |  |  |  |  |  |  |  |
|  | **M2 at the 500-m scale** |  |  |  |  |  |  |  |
|  | (R²m = 0.13; | 0,54 | 253,18 | Intercept | 17,20 | (14.22; 20.18) | - |  |
|  | R²c = 0.13) |  |  | Local farming system (:Conventional) | 0,86 | (-3.29; 5.02) | 1 |  |
|  |  |  |  | % organic farming | -1,49 | (-7.41; 4.42) | 1 |  |
|  |  |  |  | % semi-natural habitats | 2,30 | (-4.11; 8.70) | 1 |  |
|  |  |  |  | Local farming system : % organic farming | -4,72 | (-13.37; 3.94) | 1 |  |
|  |  |  |  | Local farming system : % semi-natural habitats | -1,74 | (-11.00; 6.46) | 0,77 |  |
|  |  |  |  | % organic farming : % semi-natural habitats | 0,59 | (-9.81; 11.32) | 0,78 |  |
|  |  |  |  | *pair* |  |  |  | *0* |
|  |  |  |  |  |  |  |  |  |
| **Spider species richness** | **M0** |  |  |  |  |  |  |  |
|  | (R²m = 0.06; | 0,28 | 143,76 | Intercept | 6,62 | (6.17; 7.08) | - |  |
|  | R²c = 0.24) |  |  | Field age |  |  | 0 |  |
|  |  |  |  | Vine stock density | -0,09 | (-1.36; 0.64) | 0,24 |  |
|  |  |  |  | Total TFI |  |  | 0 |  |
|  |  |  |  | Tillage intensity | 0,12 | (-0.41; 1.32) | 0,26 |  |
|  |  |  |  | Crop productivity | -0,31 | (-1.55; 0.23) | 0,47 |  |
|  |  |  |  | *pair* |  |  |  | *0,58* |
|  |  |  |  |  |  |  |  |  |
|  | **M1*** |  |  |  |  |  |  |  |
|  | (R²m = 0; | 0,04 | 143,91 | Intercept | 6,63 | (6.18; 7.07) | - |  |
|  | R²c = 0.08) |  |  | Local farming system (:Conventional) |  |  | 0 |  |
|  |  |  |  | *pair* |  |  |  | *0,38* |
|  |  |  |  |  |  |  |  |  |
|  | **M2 at the 500-m scale** |  |  |  |  |  |  |  |
|  | (R²m = 0.26; | 0,38 | 142,33 | Intercept | 6,75 | (6.20; 7.31) | - |  |
|  | R²c = 0.26) |  |  | Local farming system (:Conventional) | -0,05 | (-0.83; 0.70) | 0,72 |  |
|  |  |  |  | % organic farming | 1,16 | (0.08; 2.60) | 0,86 |  |
|  |  |  |  | % semi-natural habitats | 0,47 | (-0.47; 1.9) | 0,65 |  |
|  |  |  |  | Local farming system : % organic farming | -1,40 | (-3.54; -0.36) | 0,72 |  |
|  |  |  |  | Local farming system : % semi-natural habitats | 1,35 | (0.02; 4.13) | 0,21 |  |
|  |  |  |  | % organic farming : % semi-natural habitats | 0,19 | (-0.71; 2.51) | 0,65 |  |
|  |  |  |  | *pair* |  |  |  | *0,12* |
|  |  |  |  |  |  |  |  |  |
| **Ant species richness** | **M0** |  |  |  |  |  |  |  |
|  | (R²m = 0.07; | 0,45 | 148,96 | Intercept | 3,21 | (2.72; 3.69) | - |  |
|  | R²c = 0.16) |  |  | Field age | 0,65 | (-0.13; 1.77) | 0,79 |  |
|  |  |  |  | Vine stock density | -0,08 | (-1.50; 0.46) | 0,16 |  |
|  |  |  |  | Total TFI | -0,06 | (-1.35; 0.45) | 0,14 |  |
|  |  |  |  | Tillage intensity | 0,04 | (-0.631; 1.26) | 0,11 |  |
|  |  |  |  | Crop productivity | -0,03 | (-1.24; 0.61) | 0,11 |  |
|  |  |  |  | *pair* |  |  |  | *0,44* |
|  |  |  |  |  |  |  |  |  |
| **Total evenness** | **M0*** |  |  |  |  |  |  |  |
|  | (R²m = 0; | 0,24 | 5,25 | Intercept | 0,57 | (0.50; 0.65) | - |  |
|  | R²c = 0.07) |  |  | Field age |  |  | 0 |  |
|  |  |  |  | Vine stock density |  |  | 0 |  |
|  |  |  |  | Total TFI |  |  | 0 |  |
|  |  |  |  | Tillage intensity |  |  | 0 |  |
|  |  |  |  | Crop productivity |  |  | 0 |  |
|  |  |  |  | *pair* |  |  |  | *0,06* |
|  |  |  |  |  |  |  |  |  |
|  | **M1*** |  |  |  |  |  |  |  |
|  | (R²m = 0; | 0,19 | 5,25 | Intercept | 0,57 | (0.50; 0.65) | - |  |
|  | R²c = 0.07) |  |  | Local farming system (:Conventional) |  |  | 0 |  |
|  |  |  |  | *pair* |  |  |  | *0,06* |
|  |  |  |  |  |  |  |  |  |
|  | **M2 at the 500-m scale** |  |  |  |  |  |  |  |
|  | (R²m = 0; | 0,31 | 5,25 | Intercept | 0,57 | (0.50; 0.65) | - |  |
|  | R²c = 0.07) |  |  | Local farming system (:Conventional) |  |  | 0 |  |
|  |  |  |  | % organic farming | -0,06 | (-0.30; -0.02) | 0,36 |  |
|  |  |  |  | % semi-natural habitats |  |  | 0 |  |
|  |  |  |  | Local farming system : % organic farming |  |  | 0 |  |
|  |  |  |  | Local farming system : % semi-natural habitats |  |  | 0 |  |
|  |  |  |  | % organic farming : % semi-natural habitats |  |  | 0 |  |
|  |  |  |  | *pair* |  |  |  | *0,06* |
|  |  |  |  |  |  |  |  |  |

**TABLE S2** Responses of the natural enemy abundance, richness and evenness to environmental conditions in vineyards for the community sampled at the soil surface. All model descriptors that are reported have been obtained using the same procedure as the data reported in the Table S1. See the legend in Table S1.

| **Response variable** | **Model with R² marginal and R² conditional** | **Sum Wi** | **AIC** | **Explanatory variables selected and random term** | **Estimates** | **Confidence intervals** | **Relative variable importance** | **S.D Random term** |
| --- | --- | --- | --- | --- | --- | --- | --- | --- |
| **Total abundance** | **M0*** |  |  |  |  |  |  |  |
|  | (R²m = 0.16; | 0,00 | 500,99 | Intercept | 588,89 | (521.82; 655.95) | - |  |
|  | R²c = 0.16) |  |  | Field age | -150,43 | (-310.76; 9.90) | 1 |  |
|  |  |  |  | Vine stock density | 121,06 | (-54.41; 296.52) | 1 |  |
|  |  |  |  | Total TFI | -17,88 | (-162.13; 126.37) | 1 |  |
|  |  |  |  | Tillage intensity | 84,07 | (-67.50; 235.65) | 1 |  |
|  |  |  |  | Crop productivity | 6,49 | (-162.77; 175.75) | 1 |  |
|  |  |  |  | *pair* |  |  |  | *0* |
|  |  |  |  |  |  |  |  |  |
|  | **M1*** |  |  |  |  |  |  |  |
|  | (R²m = 0.15; | 0,00 | 532,91 | Intercept | 496,96 | (400.65; 593.26) | - |  |
|  | R²c = 0.15) |  |  | **Local farming system (:Conventional)** | **183,85** | **(47.66; 320.05)** | **1** |  |
|  |  |  |  | *pair* |  |  |  | *0* |
|  |  |  |  |  |  |  |  |  |
|  | **M2 at the 500-m scale*** |  |  |  |  |  |  |  |
|  | (R²m = 0.24; | 0,57 | 485,78 | Intercept | 506,24 | (414.87; 597.61) | - |  |
|  | R²c = 0.24) |  |  | **Local farming system (:Conventional)** | **183,21** | **(56.45425; 309.9561)** | **1** |  |
|  |  |  |  | % organic farming | -22,44 | (-204.46; 159.58) | 1 |  |
|  |  |  |  | % semi-natural habitats | -28,58 | (-238.68; 181.5) | 1 |  |
|  |  |  |  | Local farming system : % organic farming | 216,38 | (-49.31; 482.08) | 1 |  |
|  |  |  |  | Local farming system : % semi-natural habitats | 87,98 | (-178.79; 354.76) | 1 |  |
|  |  |  |  | % organic farming : % semi-natural habitats | 247,87 | (-74.55; 570.28) | 1 |  |
|  |  |  |  | *pair* |  |  |  | *0* |
|  |  |  |  |  |  |  |  |  |
| **Spider abundance** | **M0*** |  |  |  |  |  |  |  |
|  | (R²m = 0.16; | 0,00 | 458,27 | Intercept | 208,75 | (172.97; 244.53) | - |  |
|  | R²c = 0.16) |  |  | Field age | -30,10 | (-115.64; 55.44) | 1 |  |
|  |  |  |  | Vine stock density | -72,64 | (-166.25; 20.97) | 1 |  |
|  |  |  |  | Total TFI | -46,16 | (-123.13; 30.80) | 1 |  |
|  |  |  |  | Tillage intensity | 22,33 | (-58.58; 103.19) | 1 |  |
|  |  |  |  | Crop productivity | 82,03 | (-8.27; 172.33) | 1 |  |
|  |  |  |  | *pair* |  |  |  | *0* |
|  |  |  |  |  |  |  |  |  |
|  | **M1*** |  |  |  |  |  |  |  |
|  | (R²m = 0,12; | 0,00 | 486,28 | Intercept | 165,39 | (112.99; 217.78) | - |  |
|  | R²c = 0.24) |  |  | **Local farming system (:Conventional)** | **86,90** | **(16.45; 157.14)** | **1** |  |
|  |  |  |  | *pair* |  |  |  | *45,18* |
|  |  |  |  |  |  |  |  |  |
|  | **M2 at the 500-m scale*** |  |  |  |  |  |  |  |
|  | (R²m = 0.40; | 0,97 | 435,77 | Intercept | 152,14 | (109.16; 195.18) | - |  |
|  | R²c = 0.47) |  |  | **Local farming system (:Conventional)** | **78,18** | **(19.57; 135.93)** | **1** |  |
|  |  |  |  | % organic farming | -50,95 | (-136.92; 35.53) | 1 |  |
|  |  |  |  | % semi-natural habitats | -69,19 | (-168.54; 30.19) | 1 |  |
|  |  |  |  | **Local farming system : % organic farming** | **191,41** | **(69.80; 313.81)** | **1** |  |
|  |  |  |  | **Local farming system : % semi-natural habitats** | **132,64** | **(11.01; 255.95)** | **1** |  |
|  |  |  |  | **% organic farming : % semi-natural habitats** | **-214,32** | **(-375.71; -54.08)** | **1** |  |
|  |  |  |  | *pair* |  |  |  | *35,25* |
|  |  |  |  |  |  |  |  |  |
| **Ant abundance** | **M0*** |  |  |  |  |  |  |  |
|  | (R²m = 0.08; | 0,00 | 432,61 | Intercept | 169,91 | (124.58; 216.61) | - |  |
|  | R²c = 0.10) |  |  | Field age | -56,06 | (-163.71; 51.77) | 1 |  |
|  |  |  |  | Vine stock density | 104,41 | (-13.51; 219.07) | 1 |  |
|  |  |  |  | Total TFI | 9,44 | (-90.90; 108.7) | 1 |  |
|  |  |  |  | Tillage intensity | -19,92 | (-123.28; 82.88) | 1 |  |
|  |  |  |  | Crop productivity | -39,37 | (-154.34; 77.94) | 1 |  |
|  |  |  |  | *pair* |  |  |  | *20,41* |
|  |  |  |  |  |  |  |  |  |
|  | **M1*** |  |  |  |  |  |  |  |
|  | (R²m = 0,01; | 0,00 | 462,74 | Intercept | 156,67 | (91.50; 221.92) | - |  |
|  | R²c = 0.11) |  |  | Local farming system (:Conventional) | 27,66 | (-63.41; 118.97) | 1 |  |
|  |  |  |  | *pair* |  |  |  | *45,94* |
|  |  |  |  |  |  |  |  |  |
|  | **M2 at the 500-m scale*** |  |  |  |  |  |  |  |
|  | (R²m = 0.20; | 0,64 | 417,41 | Intercept | 167,17 | (107.99; 226.34) | - |  |
|  | R²c = 0.28) |  |  | Local farming system (:Conventional) | 28,81 | (-52.28; 111.03) | 1 |  |
|  |  |  |  | % organic farming | -62,43 | (-186.81; 60.25) | 1 |  |
|  |  |  |  | % semi-natural habitats | -32,19 | (-170.85; 106.51) | 1 |  |
|  |  |  |  | **Local farming system : % organic farming** | **184,40** | **(13.32; 357.04)** | **1** |  |
|  |  |  |  | Local farming system : % semi-natural habitats | 57,44 | (-118.35; 227.13) | 1 |  |
|  |  |  |  | % organic farming : % semi-natural habitats | 217,38 | (-5.65; 440.62) | 1 |  |
|  |  |  |  | *pair* |  |  |  | *43,31* |
|  |  |  |  |  |  |  |  |  |
| **Ground beetle abundance** | **M0*** |  |  |  |  |  |  |  |
|  | (R²m = 0; | 0,29 | 97,95 | Intercept | 4,62 | (4.34; 4.90) | - |  |
|  | R²c = 0.37) |  |  | Field age |  |  | 0 |  |
|  |  |  |  | Vine stock density |  |  | 0 |  |
|  |  |  |  | Total TFI |  |  | 0 |  |
|  |  |  |  | Tillage intensity |  |  | 0 |  |
|  |  |  |  | Crop productivity |  |  | 0 |  |
|  |  |  |  | *pair* |  |  |  | *0,47* |
|  |  |  |  |  |  |  |  |  |
|  | **M1*** |  |  |  |  |  |  |  |
|  | (R²m = 0; | 0,13 | 97,95 | Intercept | 4,62 | (4.34; 4.90) | - |  |
|  | R²c = 0.37) |  |  | Local farming system (:Conventional) |  |  | 0 |  |
|  |  |  |  | *pair* |  |  |  | *0,47* |
|  |  |  |  |  |  |  |  |  |
|  | **M2 at the 1000-m scale** |  |  |  |  |  |  |  |
|  | (R²m = 0; | 0,37 | 97,95 | Intercept | 4,62 | (4.34; 4.91) | - |  |
|  | R²c = 0.37) |  |  | Local farming system (:Conventional) |  |  | 0 |  |
|  |  |  |  | % organic farming |  |  | 0 |  |
|  |  |  |  | % semi-natural habitats | -0,13 | (-0.95; 0.19) | 0,33 |  |
|  |  |  |  | Local farming system : % organic farming |  |  | 0 |  |
|  |  |  |  | Local farming system : % semi-natural habitats |  |  | 0 |  |
|  |  |  |  | % organic farming : % semi-natural habitats |  |  | 0 |  |
|  |  |  |  | *pair* |  |  |  | *0,47* |
|  |  |  |  |  |  |  |  |  |
| **Rove beetle abundance** | **M0** |  |  |  |  |  |  |  |
|  | (R²m = 0.10; | 0,23 | 100,46 | Intercept | 3,52 | (3.25; 3.80) | - |  |
|  | R²c = 0.40) |  |  | Field age |  |  | 0 |  |
|  |  |  |  | Vine stock density |  |  | 0 |  |
|  |  |  |  | Total TFI | -0,25 | (-0.93; -0.01) | 0,53 |  |
|  |  |  |  | Tillage intensity | 0,21 | (-0.04; 0.94) | 0,47 |  |
|  |  |  |  | Crop productivity |  |  | 0 |  |
|  |  |  |  | *pair* |  |  |  | *0,44* |
|  |  |  |  |  |  |  |  |  |
|  | **M1*** |  |  |  |  |  |  |  |
|  | (R²m = 0,10; | 0,10 | 99,75 | Intercept | 3,27 | (2.93; 3.60) | - |  |
|  | R²c = 0.44) |  |  | **Local farming system (:Conventional)** | **0,52** | **(0.12; 0.89)** | **1** |  |
|  |  |  |  | *pair* |  |  |  | *0,47* |
|  |  |  |  |  |  |  |  |  |
|  | **M2 at the 1000-m scale** |  |  |  |  |  |  |  |
|  | (R²m = 0.20; | 0,34 | 99,49 | Intercept | 3,26 | (2.93; 3.60) | - |  |
|  | R²c = 0.46) |  |  | **Local farming system (:Conventional)** | **0,52** | **(0.13; 0.91)** | **1** |  |
|  |  |  |  | % organic farming |  |  | 0 |  |
|  |  |  |  | % semi-natural habitats | -0,35 | (-1.18; 0.03) | 0,61 |  |
|  |  |  |  | Local farming system : % organic farming |  |  | 0 |  |
|  |  |  |  | Local farming system : % semi-natural habitats | 0,06 | (-0.42; 1.15) | 0,17 |  |
|  |  |  |  | % organic farming : % semi-natural habitats |  |  | 0 |  |
|  |  |  |  | *pair* |  |  |  | *0,41* |
|  |  |  |  |  |  |  |  |  |
| **Total species richness** | **M0** |  |  |  |  |  |  |  |
|  | (R²m = 0.41; | 0,00 | 223,88 | Intercept | 24,67 | (23.03; 26.31) | - |  |
|  | R²c = 0.73) |  |  | **Field age** | **4,31** | **(1.37; 7.25)** | **1** |  |
|  |  |  |  | Vine stock density | -0,45 | (-4.35; 2.78) | 0,57 |  |
|  |  |  |  | **Total TFI** | **2,78** | **(0.43; 5.13)** | **1** |  |
|  |  |  |  | Tillage intensity | -1,82 | (-4.82; 0.45) | 0,83 |  |
|  |  |  |  | **Crop productivity** | **-3,26** | **(-6.28; -0.24)** | **1** |  |
|  |  |  |  | *pair* |  |  |  | *2,68* |
|  |  |  |  |  |  |  |  |  |
|  | **M1** |  |  |  |  |  |  |  |
|  | (R²m = 0,46; | 0,02 | 220,41 | Intercept | 26,57 | (24.63; 28.52) | - |  |
|  | R²c = 0.72) |  |  | **Field age** | **4,20** | **(1.729; 6.67)** | **1** |  |
|  |  |  |  | Total TFI | 0,28 | (-2.51; 3.66) | 0,48 |  |
|  |  |  |  | **Crop productivity** | **-2,85** | **(-5.14; -0.55)** | **1** |  |
|  |  |  |  | **Local farming system (:Conventional)** | **-3,80** | **(-6.25; -1.35)** | **1** |  |
|  |  |  |  | *pair* |  |  |  | *3,03* |
|  |  |  |  |  |  |  |  |  |
|  | **M2 at the 1000-m scale** |  |  |  |  |  |  |  |
|  | (R²m = 0.51; | 0,75 | 214,09 | Intercept | 26,10 | (24.24; 27.96) | - |  |
|  | R²c = 0.76) |  |  | **Field age** | **4,86** | **(2.34; 7.37)** | **1** |  |
|  |  |  |  | **Crop productivity** | **-2,87** | **(-5.15; -0.60)** | **1** |  |
|  |  |  |  | **Local farming system (:Conventional)** | **-3,90** | **(-5.74; -2.06)** | **1** |  |
|  |  |  |  | % organic farming | 0,39 | (-3.11; 3.89) | 1 |  |
|  |  |  |  | % semi-natural habitats | -0,42 | (-5.63; 4.79) | 1 |  |
|  |  |  |  | Local farming system : % organic farming | 0,87 | (-2.12; 5.46) | 0,52 |  |
|  |  |  |  | Local farming system : % semi-natural habitats | -6,96 | (-15.35; 1.43) | 0,45 |  |
|  |  |  |  | % organic farming : % semi-natural habitats | -0,43 | (-4.67; 2.75) | 1 |  |
|  |  |  |  | *pair* |  |  |  | *2,7* |
|  |  |  |  |  |  |  |  |  |
| **Spider species richness** | **M0** |  |  |  |  |  |  |  |
|  | (R²m = 0; | 0,41 | 92,81 | Intercept | 3,33 | (3.11; 3.55) | - |  |
|  | R²c = 0) |  |  | Field age | 0,05 | (-0.15; 0.75) | 0,17 |  |
|  |  |  |  | Vine stock density | 0,11 | (-0.01; 0.97) | 0,22 |  |
|  |  |  |  | Total TFI |  |  | 0 |  |
|  |  |  |  | Tillage intensity |  |  | 0 |  |
|  |  |  |  | Crop productivity | -0,19 | (-0.98; 0.07) | 0,43 |  |
|  |  |  |  | *pair* |  |  |  | *0* |
|  |  |  |  |  |  |  |  |  |
| **Ant species richness** | **M0*** |  |  |  |  |  |  |  |
|  | (R²m = 0; | 0,37 | 98,30 | Intercept | 3,60 | (3.33; 3.87) | - |  |
|  | R²c = 0) |  |  | Field age |  |  | 0 |  |
|  |  |  |  | Vine stock density |  |  | 0 |  |
|  |  |  |  | Total TFI |  |  | 0 |  |
|  |  |  |  | Tillage intensity |  |  | 0 |  |
|  |  |  |  | Crop productivity |  |  | 0 |  |
|  |  |  |  | *pair* |  |  |  | *0* |
|  |  |  |  |  |  |  |  |  |
| **Ground beetle species richness** | **M0** |  |  |  |  |  |  |  |
|  | (R²m = 0,25; | 0,55 | 148,62 | Intercept | 5,61 | (5.10; 6.12) | - |  |
|  | R²c = 0,43) |  |  | **Field age** | **1,17** | **(0.20; 2.13)** | **1** |  |
|  |  |  |  | Vine stock density | 0,02 | (-1.08; 1.32) | 0,13 |  |
|  |  |  |  | Total TFI | 0,08 | (-0.35; 1.30) | 0,17 |  |
|  |  |  |  | Tillage intensity | -0,13 | (-1.50; 0.32) | 0,23 |  |
|  |  |  |  | Crop productivity | -0,88 | (-1.95; -0.07) | 0,87 |  |
|  |  |  |  | *pair* |  |  |  | *0,7* |
|  |  |  |  |  |  |  |  |  |
| **Rove beetle species richness** | **M0** |  |  |  |  |  |  |  |
|  | (R²m = 0; | 0,40 | 58,45 | Intercept | 2,49 | (2.31; 2.67) | - |  |
|  | R²c = 0,48) |  |  | Field age | 0,06 | (0.01; 0.565) | 0,21 |  |
|  |  |  |  | Vine stock density | 0,09 | (-0.01; 0.58) | 0,3 |  |
|  |  |  |  | Total TFI | 0,08 | (0.01; 0.48) | 0,32 |  |
|  |  |  |  | Tillage intensity |  |  |  |  |
|  |  |  |  | Crop productivity |  |  |  |  |
|  |  |  |  | *pair* |  |  |  | *0,33* |
|  |  |  |  |  |  |  |  |  |
| **Total evenness** | **M0*** |  |  |  |  |  |  |  |
|  | (R²m = 0; | 0,13 | -57,62 | Intercept | 0,69 | (0.66; 0.72) | - |  |
|  | R²c = 0) |  |  | Field age |  |  | 0 |  |
|  |  |  |  | Vine stock density |  |  | 0 |  |
|  |  |  |  | Total TFI |  |  | 0 |  |
|  |  |  |  | Tillage intensity |  |  | 0 |  |
|  |  |  |  | Crop productivity |  |  | 0 |  |
|  |  |  |  | *pair* |  |  |  | *0* |
|  |  |  |  |  |  |  |  |  |
|  | **M1** |  |  |  |  |  |  |  |
|  | (R²m = 0,21; | 0,28 | -59,45 | Intercept | 0,72 | (0.67; 0.78) | - |  |
|  | R²c = 0.31) |  |  | Local farming system (:Conventional) | -0,07 | (-0.15; -0.04) | 0,71 |  |
|  |  |  |  | *pair* |  |  |  | *0,03* |
|  |  |  |  |  |  |  |  |  |
|  | **M2 at the 500-m scale** |  |  |  |  |  |  |  |
|  | (R²m = 0,21; | 0,30 | -59,45 | Intercept | 0,72 | (0.67; 0.78) | - |  |
|  | R²c = 0.31) |  |  | Local farming system (:Conventional) | -0,07 | (-0.15; -0.04) | 0,71 |  |
|  |  |  |  | % organic farming |  |  | 0 |  |
|  |  |  |  | % semi-natural habitats |  |  | 0 |  |
|  |  |  |  | Local farming system : % organic farming |  |  | 0 |  |
|  |  |  |  | Local farming system : % semi-natural habitats |  |  | 0 |  |
|  |  |  |  | % organic farming : % semi-natural habitats |  |  | 0 |  |
|  |  |  |  | *pair* |  |  |  | *0,03* |
|  |  |  |  |  |  |  |  |  |

**FIGURE S1** Relationships between proportions of forests, meadows and semi-natural habitats (i.e. forests plus meadows) at the 1000-m scale.


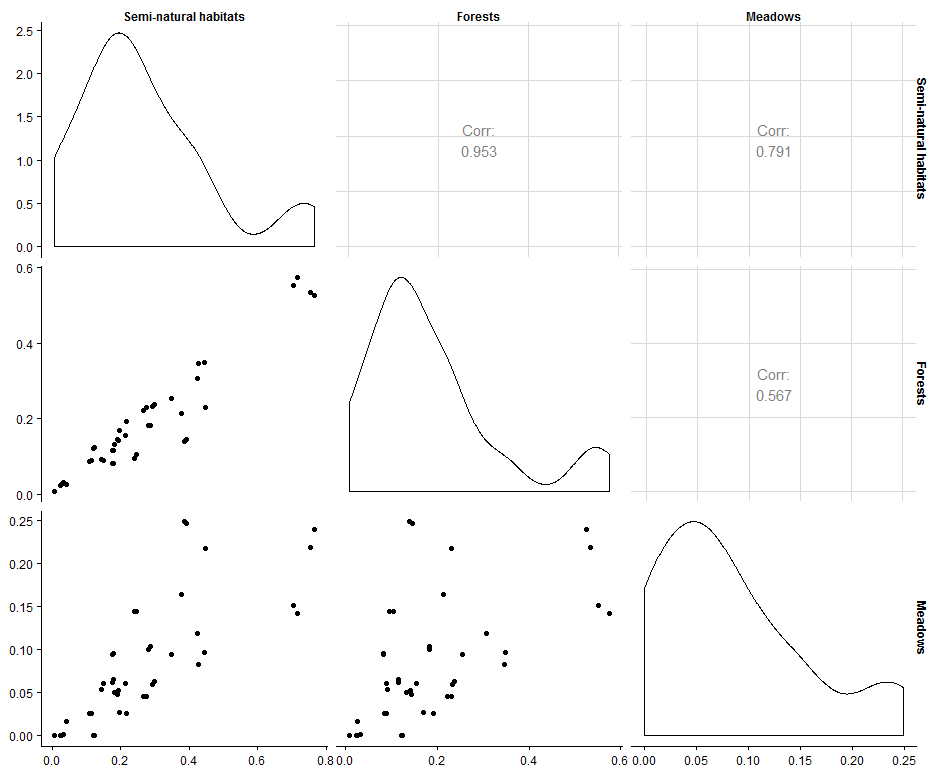


**FIGURE S2** Correlation matrix between all covariates included in the models
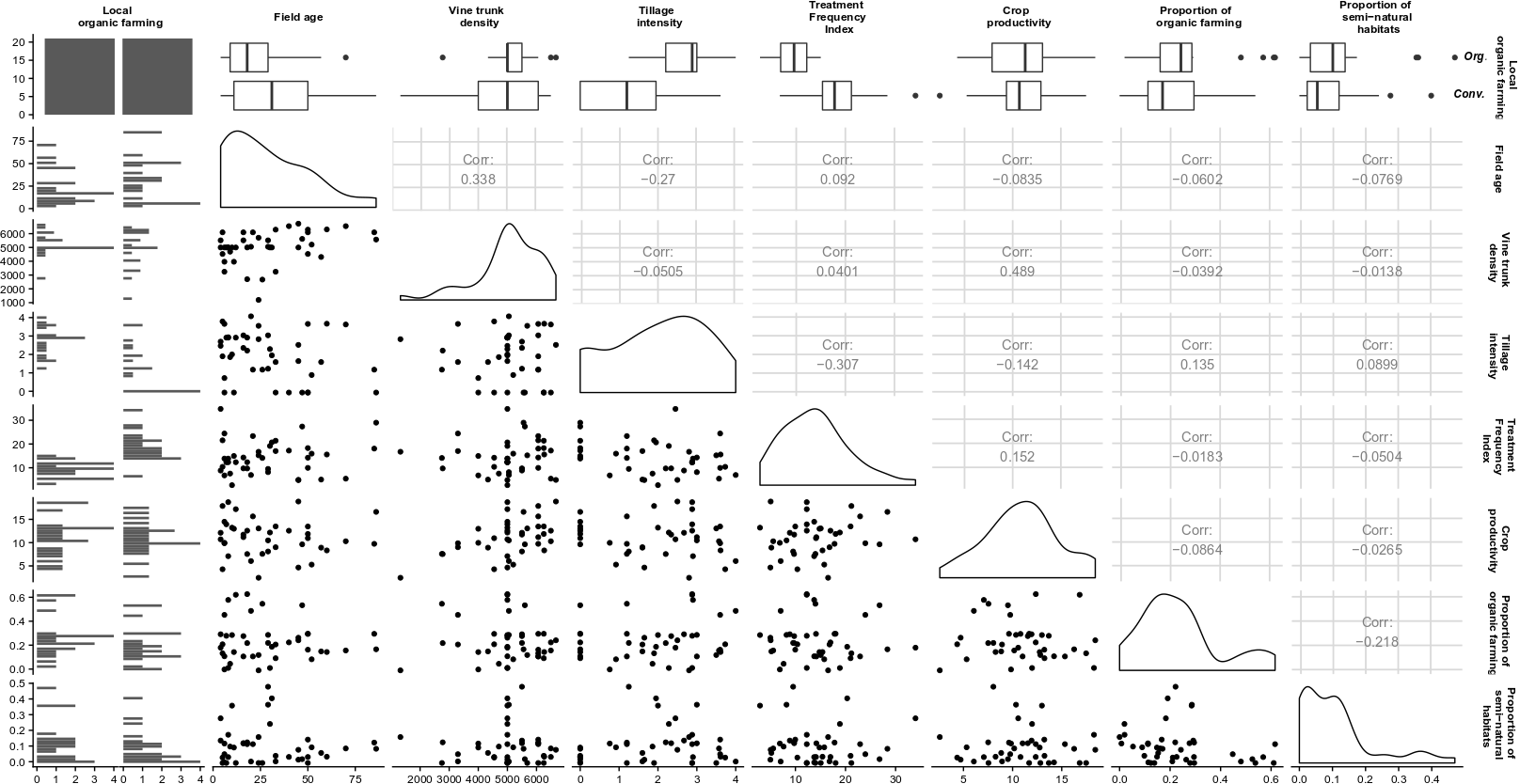


**FIGURE S3** Probability of each scale to explain the response variables. For each response variable, we recalculated the Akaike weights among all of the models from the four different sets ('M0', 'M1' and both 'M2' obtained using multimodel inference). We, therefore, estimated the relative importance of each level of complexity for a given response variable that gave us the "best scale" of response (figure S2). The sum of the Akaike weights ("Sum Wi") of the models obtained at the best scale provided the model’s probability of being the top model across all of the scales.
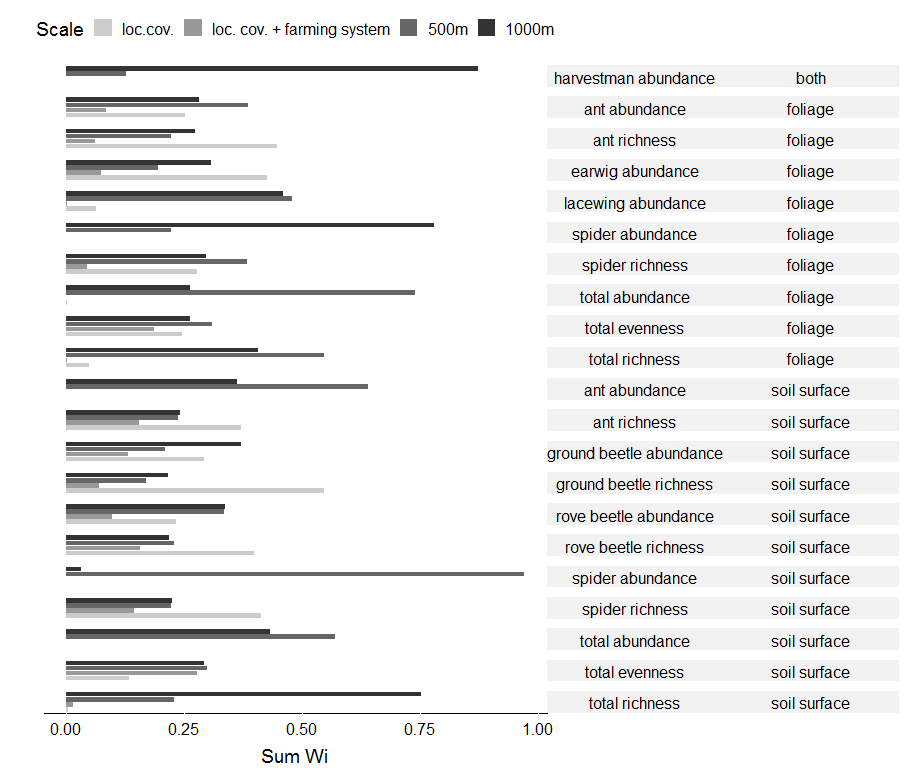


**FIGURE S4** Abundances of the 30 dominant species of the above-ground community in organic and conventional fields.


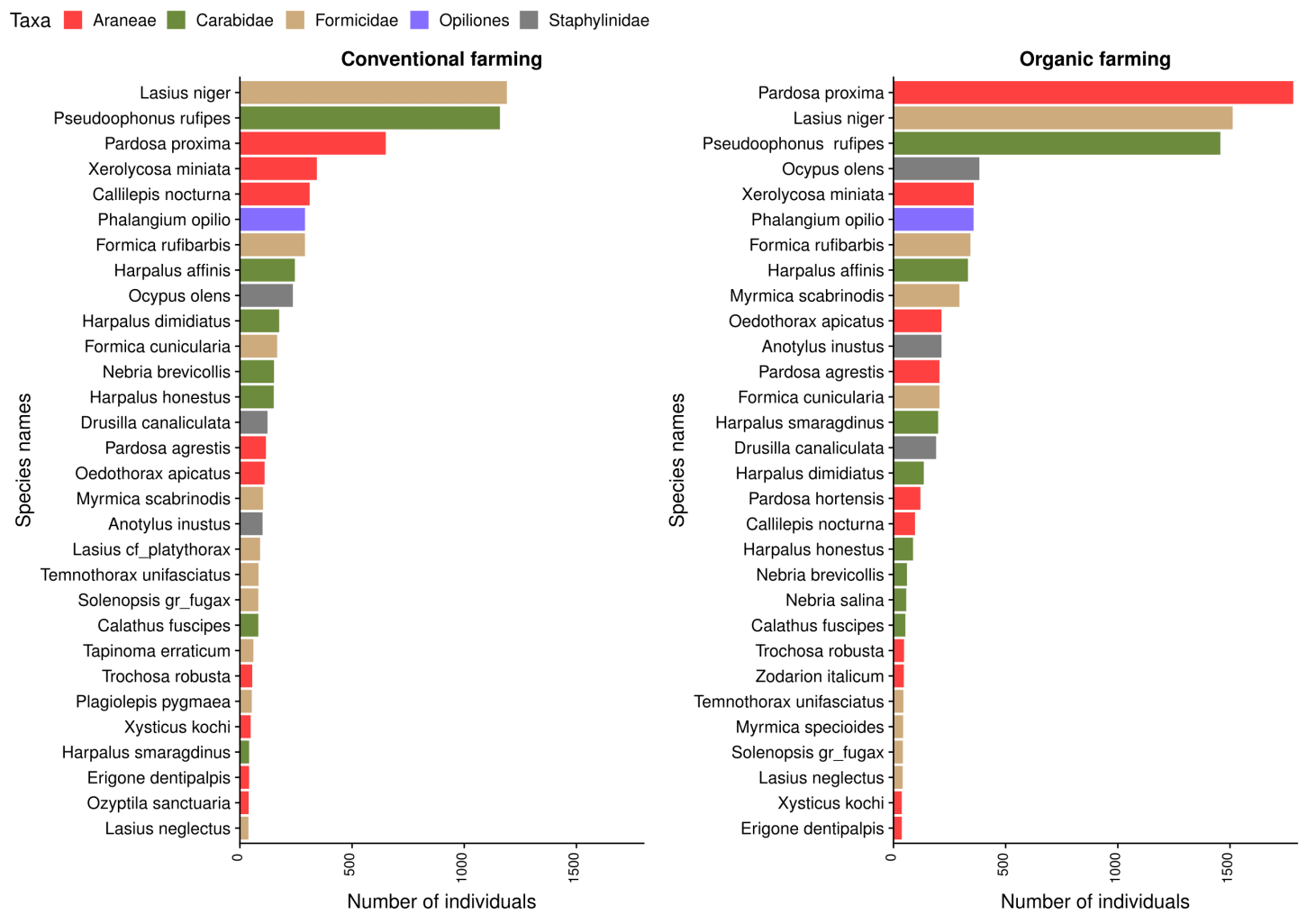

Supplement: Supplementary file 1 [file ECE3-9-13532-s001.docx]
